# Pattern Recognition and Characterization of Upper Limb Neuromuscular Dynamics during Driver-Vehicle Interactions

**DOI:** 10.1016/j.isci.2020.101541

**Published:** 2020-09-07

**Authors:** Yang Xing, Chen Lv, Yifan Zhao, Yahui Liu, Dongpu Cao, Sadahiro Kawahara

**Affiliations:** 1 School of Mechanical and Aerospace Engineering, Nanyang Technological University, Singapore 639798, Singapore; 2 School of Aerospace, Transport and Manufacturing, Cranfield University, Bedford MK43 0AL, UK; 3 School of Vehicle and Mobility, Tsinghua University, Beijing 100084, China; 4 Mechanical and Mechatronics Engineering, University of Waterloo, ON N2L 3G1, Canada; 5 Research and Development Headquarters, JTEKT Corporation, Kashihara, Nara 634-8555, Japan

**Keywords:** Kinesiology, Human-Centered, Computing, Human-Computer Interaction, Interaction Design

## Abstract

In this work, pattern recognition and characterization of the neuromuscular dynamics of driver upper limb during naturalistic driving were studied. During the human-in-the-loop experiments, two steering tasks, namely, the passive and active steering tasks, were instructed to be completed by the subjects. Furthermore, subjects manipulated the steering wheel with two distinct postures and six different hand positions. The neuromuscular dynamics of subjects' upper limb were measured using electromyogram signals, and the behavioral data, including the steering torque and steering angle, were also collected. Based on the experimental data, patterns of muscle activities during naturalistic driving were investigated. The correlations, amplitudes, and responsiveness of the electromyogram signals, as well as the smoothness and regularity of the steering torque were discussed. The results reveal the mechanisms of neuromuscular dynamics of driver upper limb and provide a theoretical foundation for the design of the future human-machine interface for automated vehicles.

## Highlights

• Driver upper limb neuromuscular dynamics were studied
• The impacts of different hand postures on driving performance were analyzed
• Correlation, amplitude, and time delay of the neuromuscular dynamics were studied
• Active and passive steering behaviors of naturalistic driving were analyzed

## Graphical Abstract

**Figure:**
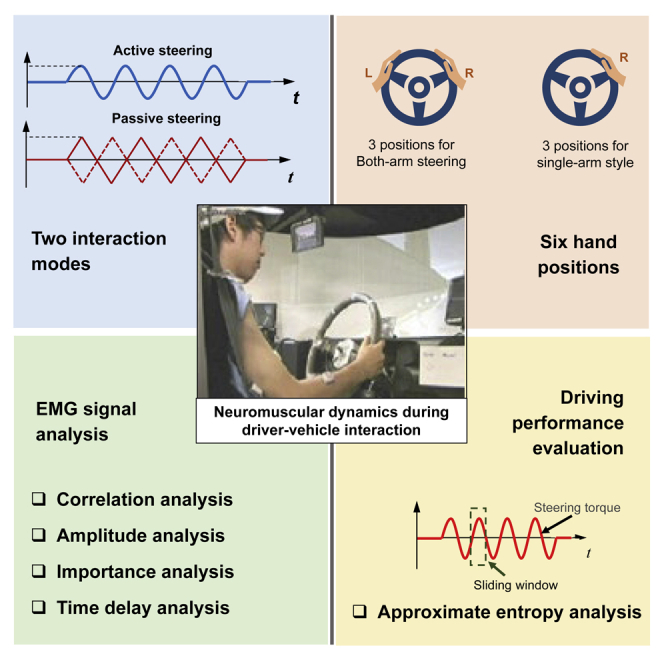

## Introduction

Since the modern vehicle was first invented in the nineteenth century, the modality and the quality of people's lives have been changed dramatically by road mobility (Smith, 1936). However, although automobiles have already been massively deployed for over a hundred years, the mechanisms of driver behaviors are still not fully understood. It is known that human drivers play a critical role during driving in terms of vehicle safety, ride comfort, and energy efficiency. In recent years, automated vehicles have gained increasing attention from both academia and the industrial sector (National Highway Traffic Safety Administration; 2017). Automated vehicles are considered a promising alternative to replace human-driven vehicles, with the aim of reducing casualties, improving traffic efficiency, and lowering human driver workloads (Kyriakidis et al., 2015; Bansal et al., 2016; Lv et al., 2017; Nunes et al., 2018; Xing et al., 2020). Although the capabilities in highly and even fully automated driving have continuously increased, unresolved problems still exist due to strong uncertainties and complex driver-vehicle interactions. In this context, one of the critical issues is to guarantee safe and smooth interactions between automatic functionality and manual driving (Saleh et al., 2013; Erlien et al., 2015; Eriksson and Stanton, 2017). This challenge requires an in-depth understanding of driver behavior and the design of human-machine collaboration.

Although some studies about muscle characteristics during vehicle steering have been investigated since the 1970s, the functions and patterns of people's muscles during naturalistic driving, and particularly when interacting with automated driving vehicle, are still unclear, especially from the biomechanical perspective. Driver neuromuscular studies have dominated the development of the collaborative steering system design of automated driving vehicles (Hallé and Chaib-draa, 2005; Fuchs et al., 2007; Nguyen et al., 2017). The neuromuscular dynamics of an upper limb were widely applied to the development of shared steering systems, haptic take-over systems, steering assistance systems, and driving fatigue detection systems (Pick and Cole, 2008; Nash and Cole, 2016; Fan et al., 2019). The lateral driver model that used the queuing network and driver neuromuscular dynamic model significantly improved the vehicle lateral control performance with high vehicle speed (Bi et al., 2015). The co-contraction features of the muscle activity supported an optimal control strategy for the muscle reflex system to assist the steering- and path-following tasks (Pick and Cole, 2006). Furthermore, a neuromuscular model considering co-activation could be represented as a combination of feedforward control and feedback control (Hoult and Cole, 2008). The model could generate feedforward control signals while minimizing the feedback error to the assistant steering control system. Moreover, the neuromuscular model was also adapted to the increases of reflex delay, which were efficient in the control of the system stability. In terms of a haptic take-over system, the driver neuromuscular dynamics differed significantly from the active steering and passive steering, for which the stiffness coefficient in the passive steering modes was much larger than that in the active steering modes (Lv et al., 2018). The electromyogram (EMG) enabled the neuromuscular analysis to provide an objective evaluation method for the steering comfort by modeling the relationship between the EMG signals and the steering comfort rate with an artificial neural network (Liu et al., 2017). Preliminary studies on the driver workload estimation using EMG signals have suggested that muscle activities depend on the steering direction (Hayama et al., 2013). The anterior deltoid, pectoralis clavicular, and infraspinatus of the right arm are responsible for the counterclockwise steering, whereas the triceps long head muscle is the agonist for the clockwise steering for the single right arm steering posture. Moreover, with the driving posture using both hands, the muscles from the right and left limbs also respond differently to the steering direction. These preliminary studies have shown that the driver neuromuscular dynamics play a critical role in the future design of intelligent vehicles and the corresponding interaction system. However, one of the limitations of these findings is the lack of comprehensive analysis by integrating both the correlation analysis results and the observed muscle amplitudes, which is an important cue for muscle importance analysis.

The analysis of the neuromuscular dynamics of an upper limb is also critical to the development of the shared control and collaborative driving mode for automated driving vehicles (Abbink et al*.*, 2011a, 2011b). The knowledge of the muscle activities and responses to the control signals from a machine enables the machine to understand intentions, capabilities, and stabilities with human steering behavior. Existing studies mainly focus on the modeling of lateral control systems such as the lane departure assistant and lane-keeping assistant systems by integrating neuromuscular dynamics such as co-contraction into consideration (Pick and Cole, 2007; Abbink et al., 2011a, 2011b). However, it is not clear how the neuromuscular dynamics are influenced by the steering task and how the neuromuscular behaviors determine and dominate the steering input. It is essential to answer these questions and to study the mechanisms of neuromuscular dynamics during different steering tasks so that an efficient collaborative driving system can be developed for next-generation automated vehicles.

**Figure 1:**
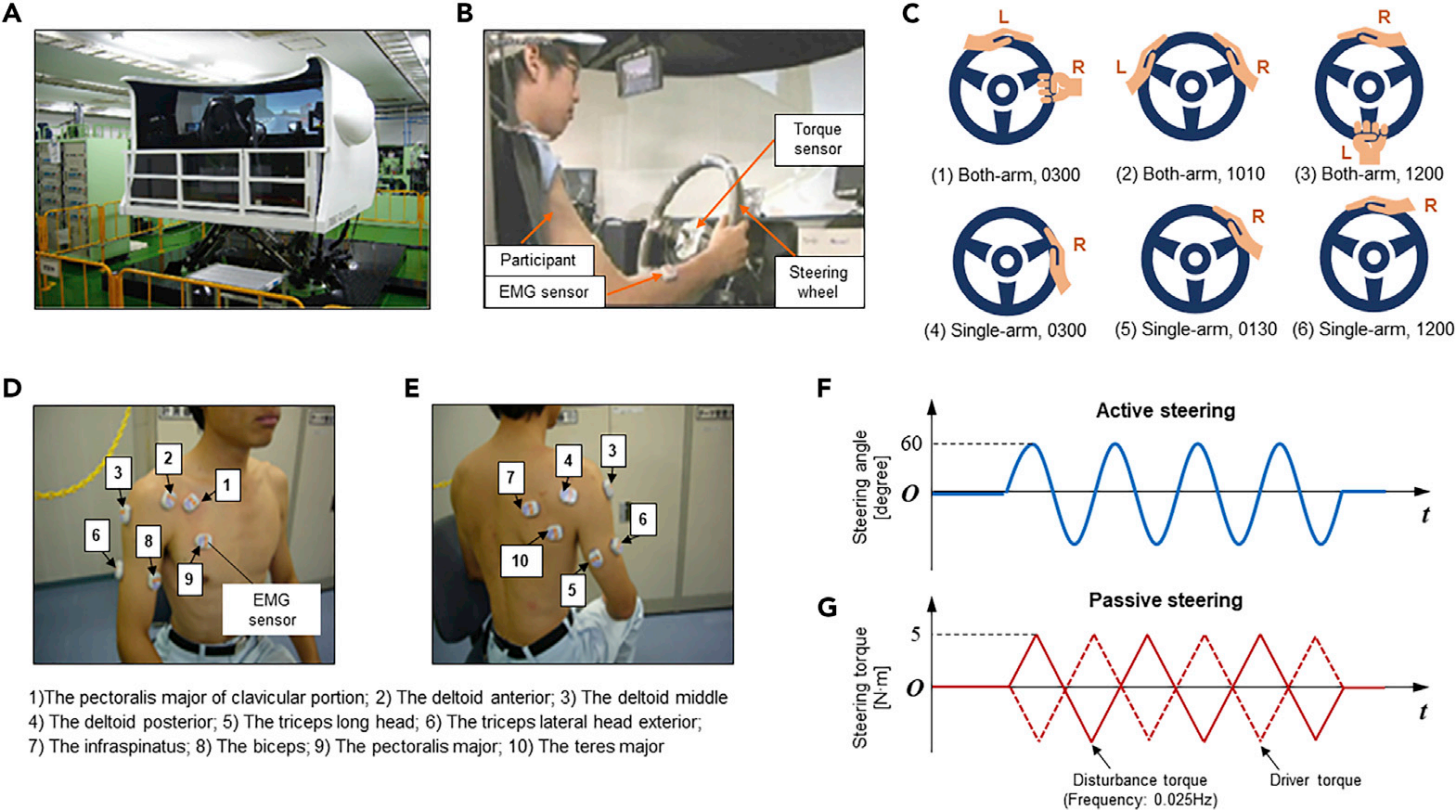
Experimental Setup (A) The experimental platform used in this study was a human-in-the-loop driving simulator. (B) View from inside the driving simulator. Key components used in the experiment mainly include the EMG sensors, the steering wheel, and the torque and angle sensor. (C), Illustration of the six distinct hand positions. As shown in the subplots of (1), (2), and (3), the driver operates the steering wheel with both arms. The three gripping positions are the 3 o'clock position (denoted as 0300), 10:10 position (denoted as 1010), and 12 o'clock position (denoted as 1200), respectively. Also, as shown in the subplots (4), (5), and (6), the driver operates the steering wheel using the right arm only, and the left hand is held away from the steering wheel. The three gripping positions are the 3 o'clock position (denoted as 0300), 1:30 position (denoted as 0130), and 12 o'clock position (denoted as 1200), respectively. (D) EMG measurement regions on the right arm. (E) EMG measurement regions on the right arm. (F) The scenario of the active steering task. (G) The scenario of the passive steering task. See also Figure S1, Tables S1 and S2.

Considering these challenges, in this work, driver neuromuscular dynamics were studied with consideration of different driving postures and steering objectives. Specifically, we analyzed the different muscle responses to two different steering tasks, namely, the active steering and passive steering. The basic posture of an example participant is shown in Figure S1. The active steering required the drivers to steer the wheel independently and to follow the pre-defined target object. However, the drivers needed to hold the steering wheel steadily to overcome the disturbance steering torque from the machine actuator in the passive steering mode. Moreover, three hand positions and their influences on the neuromuscular dynamics and steering performance were designed. Experiments were conducted in a driving simulator. A total of 42 participants were involved in the experiment. The testing scenarios are shown in Figure 1. Detailed specifications of the measured signals can be found in Tables S1 and S2. In summary, the following four basic but critical aspects of this study were investigated in this study.

1) A correlation analysis between the EMG signals and the steering torque was performed with consideration of the steering directions, steering objectives, and steering postures.
2) The amplitude analysis of the EMG signals was performed to evaluate the steering effort. A larger amplitude indicates a more significant response of an EMG signal to the steering task. Besides, different steering scenarios were considered. Moreover, a comprehensive analysis that integrates the results of correlation and amplitude was conducted to explore the key muscles during naturalistic driving.
3) We studied the time delay characteristics between the EMG signals and steering toque based on the cross-correlation analysis, which contributed to the development of the steering torque prediction system based on the neuromuscular dynamics.
4) We analyzed the impact of the steering postures on the driving performance. The driving and steering performance was characterized by the smoothness and regularity of the steering torque. The approximate entropy and sliding standard deviation tests were applied for the steering performance evaluation.

## Results

In this work, the patterns of the correlation between the upper limb neuromuscular dynamics of humans and the output steering torque were studied, as well as human performance during normal driving tasks. Driving experiments for active and passive steering maneuvers using both-arm and single-arm arrangements with the different hand gripping positions were conducted and analyzed. The following four aspects, namely, (1) the correlation between the EMG signals measured from the drivers' upper limbs and the steering torques, (2) the quantitative evaluation of the strength and importance of the EMG signals for different driving modes, (3) the time delay of the steering torque after the generation of the EMG signals, and (4) the performance and smoothness of human drivers during the various steering experiments were investigated. In the single-arm steering scenarios, 10 different neuromuscular signals denoted as MS1–MS10 were measured from the right upper limb. These 10 signals were the pectoralis major of clavicular portion, the deltoid anterior, the deltoid middle (lateral), the deltoid posterior, the triceps long head, the triceps lateral head exterior, the infraspinatus, the biceps, the pectoralis major, and the teres major. For the steering experiments with the both-arm arrangement, 10 EMG signals, denoted as MB1–MB10, were detected from both the right and left upper limbs. They were the pectoralis major of the clavicular portion, the deltoid anterior, the deltoid posterior, the triceps long head, and the teres major of the right arm and the left arm. In total, there were 42 participants in the experiments. Among these participants, 20 were randomly assigned to conduct the single-arm experiments, and the rest of the 22 participants were engaged in the both-arm steering experiments. The detailed experimental results are described as follows. The values are presented in the form of the mean ± SD.

### Correlation between the EMG Signals and the Steering Torque

**Figure 2:**
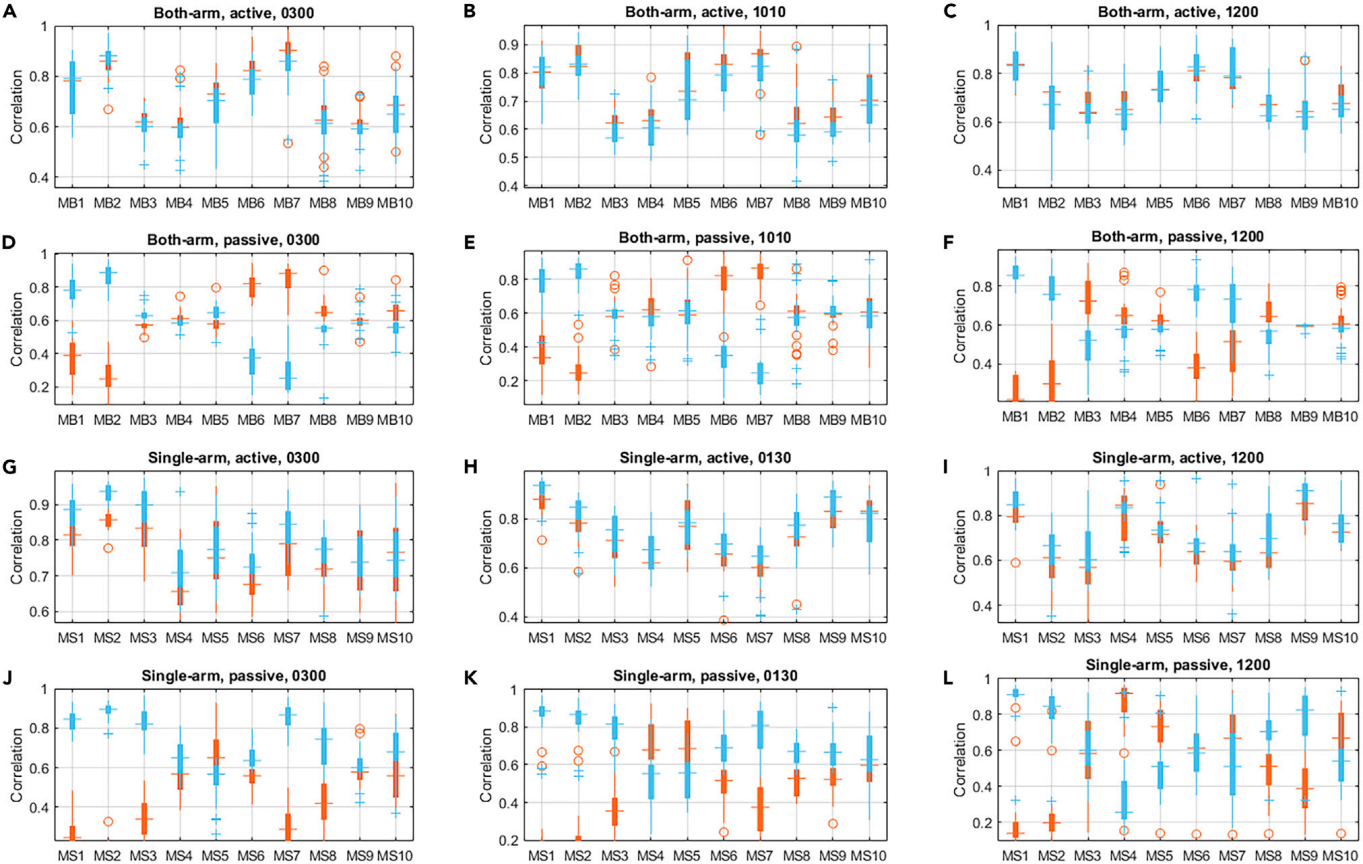
The Results of the Cross-correlation between Different EMG Signals Were Measured from the Upper Limb and the Steering Torque Signal The cross-correlation results were sensitive to the steering directions. The red boxplots indicate the correlations between the neuromuscular signals and the corresponding steering torque with the clockwise direction, whereas the light blue boxplots indicate the results of the steering torque in the counterclockwise direction. (A) The cross-correlation results of both-arm experiments under active steering with hand position of 3 o'clock. (B) The results of both-arm, active steering with hand position of 10:10. (C) The results of both-arm, active steering with hand position of 12 o'clock. (D) The results of both-arm, passive steering with hand position of 3 o'clock. (E) The results of both-arm, passive steering with hand position of 10:10. (F) The results of both-arm, passive steering with hand position of 12 o'clock. (G) The cross-correlation results of single-arm experiments under active steering with hand position of 3 o'clock. (H) The results of single-arm, active steering with hand position of 1:30. (I) The results of single-arm, active steering with hand position of 12 o'clock. (J) The results of single-arm, passive steering with hand position of 3 o'clock. (K) The results of single-arm, passive steering with hand position of 1:30. (L) The results of single-arm, passive steering with hand position of 12 o'clock. See also Tables S3–S10.

The correlation between the EMG signals and the steering torque was analyzed for the both-arm and single-arm driving experiments with respect to different hand gripping positions. The motivation for this analysis was the determination of which muscular signals were significantly relevant to the steering tasks under different conditions. The results of the cross-correlation analysis for the both-arm and single-arm steering scenarios are shown in Figure 2. Based on the results, it could be found that different muscles exhibited different correlations to the steering tasks (active/passive steering). In addition, the different muscles were sensitive to the steering directions (counterclockwise/clockwise), hand gripping positions, and the number of arms that were used to control the steering wheel (single-arm or both-arm). Key results are reported as follows, and detailed statistics can be found in Tables S3–S10.

The top two rows in Figure 2 show the cross-correlation results for the both-arm steering experiments. Specifically, for the both-arm experiments with the active steering mode, the correlation of the 10 selected muscles with respect to different hand positions and steering directions showed consistent patterns. The correlation values of the MB1, MB2, MB6, and MB7 were all larger than 0.75, showing a strong correlation to the corresponding steering activities with hand positions of 3 o'clock and 10:10 o'clock. Similar patterns could be found in the 12 o'clock scenarios, while one outlier was that the MB2 on the right arm was not strongly correlated with the driving activities in both the clockwise (0.683 ± 0.143) and counterclockwise (0.654 ± 0.139) directions.

However, for the steering experiments with the single-arm (the right arm) arrangement, the observed patterns of the cross-correlation results were different for the three different hand positions, as shown in the bottom two rows in Figure 2. Specifically, for the scenario of active steering with the 3 o'clock hand position, six muscles, namely, the MS1, MS2, MS3, MS5, MS7, and MS10, showed strong correlations (correlation larger than 0.75) to the steering in both the clockwise and counterclockwise directions. The first three muscles showed higher correlations. The MS1 achieved the correlation values of 0.815 ± 0.054 and 0.878 ± 0.050 in both the clockwise and counterclockwise directions, respectively. High values of correlation could also be observed from the results of MS2 and MS3. For the single-arm experiments with the hand position of 1:30 o'clock, similar patterns could be observed, although the results of MS3 showed some exceptional phenomena. In addition, in the 1:30 o'clock scenarios, the MS9 generated the values at 0.821 ± 0.071 and 0.865 ± 0.070 in the clockwise and the counterclockwise directions, indicating strong correlations to the steering tasks. Last, with the hand position of 12 o'clock, three muscles, namely, MS1, MS4, and MS9, revealed consistently strong correlations to the steering maneuvers. However, the overall patterns of the correlation pattern for the 12 o'clock hand position were quite different from the other two cases with the 3 o'clock and 1:30 o'clock hand positions.

In the comparison of the results of the two steering tasks, i.e., the active steering and passive steering, an obvious difference was the directional dependence. For the active steering experiments, no significant difference regarding clockwise and counterclockwise directions could be observed (p > 0.05). However, for the passive steering experiments, significant differences of the correlations were observed with consideration of steering directions. For example, in the both-arm passive steering experiments with the hand positions, the correlation values of MB1 and MB2 were 0.766 ± 0.126 and 0.840 ± 0.082 for the hand position of 3 o'clock and 0.779 ± 0.096 and 0.865 ± 0.071 for the 10:10 o'clock hand position, indicating strong correlations to the counterclockwise direction. The muscle activities showed weak correlations to the clockwise direction, reflected by the correlation values of MB1 and MB2 being at 0.373 ± 0.113 and 0.264 ± 0.099 for the 3 o'clock position, and 0.354 ± 0.124 and 0.260 ± 0.104 for the 10:10 o'clock position. Similar directional-dependent results could also be found in the correlation results of MB1, MB2, MB3, MB6, and MB7.

### Amplitude Analysis of the EMG Signals

**Figure 3:**
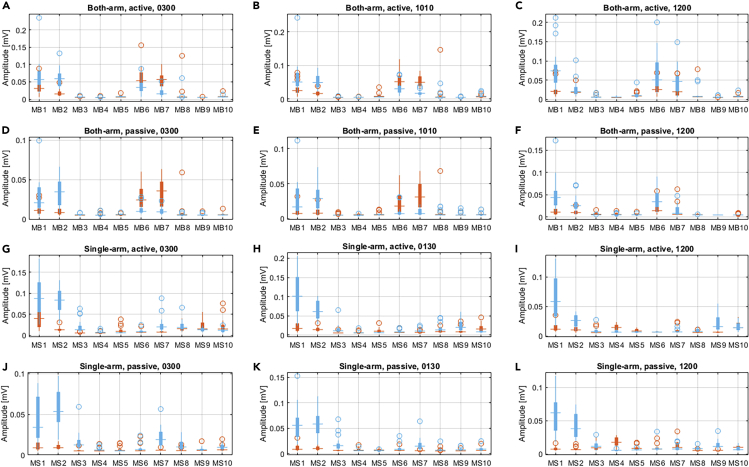
Directional-Oriented Amplitude Statistics for Different Driving Modes with Respect to the Clockwise and Counterclockwise Steering Directions The orange boxes exhibit the amplitude statistics for the clockwise steering, and the light blue boxes show the statistics with respect to the counterclockwise steering. (A) The cross-correlation results of both-arm, active steering with hand position of 3 o'clock. (B) The results of both-arm, active steering with hand position of 10:10. (C) The results of both-arm, active steering with hand position of 12 o'clock. (D) The results of both-arm, passive steering with hand position of 3 o'clock. (E) The results of both-arm, passive steering with hand position of 10:10. (F) The results of both-arm, passive steering with hand position of 12 o'clock. (G) The cross-correlation results of single-arm, active steering with hand position of 3 o'clock. (H) The results of single-arm, active steering with hand position of 1:30. (I) The results of single-arm, active steering with hand position of 12 o'clock. (J) The results of single-arm, passive steering with hand position of 3 o'clock. (K) The results of single-arm, passive steering with hand position of 1:30. (L) The results of single-arm, passive steering with hand position of 12 o'clock. See also Figure S2, Tables S11–S16.

This section describes how the amplitude of the EMG signals for different participants was analyzed under each testing scenario. The direction-dependent amplitudes of the EMG signals were statistically analyzed, as illustrated in Figure 3. In general, based on the results, it could be found that for experiments with both single-arm and both-arm arrangements, the pectoralis major of the clavicular portion and the deltoid anterior muscles generated significantly higher responses than the other muscles in terms of the amplitude value. In addition, the amplitude statistics of the EMG signals without consideration of the steering directions are reported in Figure S2. Key results are reported as follows, and detailed statistics can be found in Tables S11–S16 in the supplemental file.

As shown in Figure 3, in the steering experiments with a single right arm in the counterclockwise direction, the amplitudes of the EMG signals were significantly larger than those in the clockwise steering experiments, for both the active and passive steering cases. Moreover, the MS1, i.e., the pectoralis major, and the MS2, the deltoid anterior, of the right arm generated significant larger amplitudes than the other EMG signals did. In contrast, for the both-arm driving experiments with both active and passive steering modes, a consistent pattern for the amplitudes of the EMG signals could be found. We observed that the pectoralis major and the deltoid anterior of the left and right arms had a different response to the steering maneuvers in terms of the correlation values. Specifically, the MB1 and MB2, i.e., the pectoralis major and the deltoid anterior of the right arm, showed significant responses to the counterclockwise steering activities with the hand positions of 3 o'clock and 10:10 o'clock for both active and passive steering. However, the corresponding muscles MB6 and MB7, i.e., the pectoralis major and the deltoid anterior on the left arm, were more relevant to the clockwise steering maneuvers. In addition, it was interesting to see that the amplitudes of MB6 and MB7 during counterclockwise steering were larger than those in the clockwise direction. This was opposite to the patterns of the MB1 and MB2 mentioned above. However, it is also interesting to see that such opposite patterns were not observed with the hand positions of 12 o'clock.

**Figure 4:**
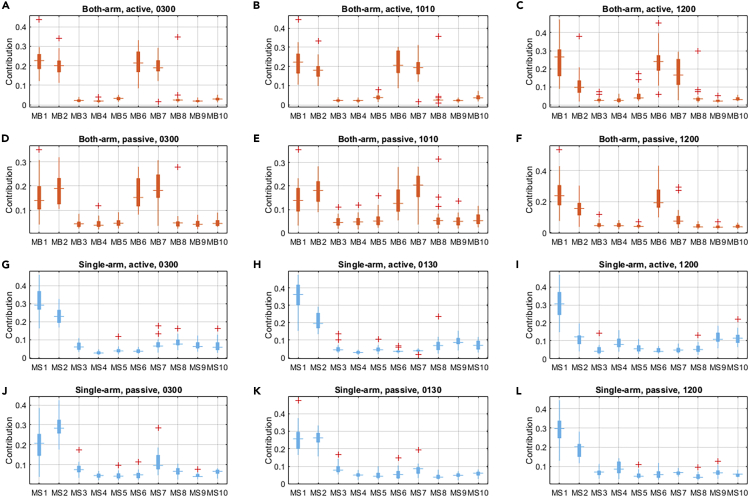
Overall Muscle Contribution Statistics for Different Driving Modes The orange boxes exhibit the contribution statistics for the both-arm driving mode, and the light blue boxes show the statistics with respect to the single-arm driving mode. (A) The cross-correlation results of both-arm, active steering with hand position of 3 o'clock. (B) The results of both-arm, active steering with hand position of 10:10. (C) The results of both-arm, active steering with hand position of 12 o'clock. (D) The results of both-arm, passive steering with hand position of 3 o'clock. (E) The results of both-arm, passive steering with hand position of 10:10. (F) The results of both-arm, passive steering with hand position of 12 o'clock. (G) The cross-correlation results of single-arm, active steering with hand position of 3 o'clock. (H) The results of single-arm, active steering with hand position of 1:30. (I) The results of single-arm, active steering with hand position of 12 o'clock. (J) The results of single-arm, passive steering with hand position of 3 o'clock. (K) The results of single-arm, passive steering with hand position of 1:30. (L) The results of single-arm, passive steering with hand position of 12 o'clock. See also Tables S17–S20.

Next, the contribution ratio of each muscle to the corresponding steering activity was further analyzed based on the integration of the correlation and the amplitude of each EMG signal in each direction (for the detailed computation method, refer to the section Amplitude Analysis in the Transparent Methods). The overall contribution statistics are numerically described and visualized in Figure 4. For the both-arm experiments based on the contribution analysis of the data with both active and passive steering, patterns that were consistent with the correlation analysis could be found. It was shown that MB1, MB2, MB6, and MB7 for the left and right arms played the most important roles with the both-arm driving condition. Although some muscles such as the MB3 and MB5 showed a strong correlation to the steering torque, their amplitudes and the overall contributions were not large enough to be viewed as important muscles. Similar results could be found in the single-arm steering mode. Specifically, the MS1 and MS2 were the two most important muscles in both the active and passive steering maneuvers. Moreover, according to the statistics, most of the contribution ratios of the measured EMG signals had median values within 0.05 and 0.15. Detailed statistical analysis can be found in Tables S17–S20 in the supplemental file.

### Time Delay between the EMG Signals and the Steering Torque

The time delay between the neuromuscular signals and the generated steering torque signal was analyzed in this part. Specifically, the four key muscles (MB1, MB2, MB3, and MB4) for the both-arm steering mode and the two key muscles (MS1 and MS2) for the single-arm steering mode were selected for the analysis. The time delay with the maximum absolute correlation could be measured when the two signals were best aligned. A negative value for the time delay indicated that the activity of the specific muscle led to the generation of the steering torque. If the time delay was positive, then the muscle signal lagged behind the steering torque due to the co-contraction mechanism of the muscles. Key results are reported as follows, and detailed statistics can be found in Tables S21–S24.

**Figure 5:**
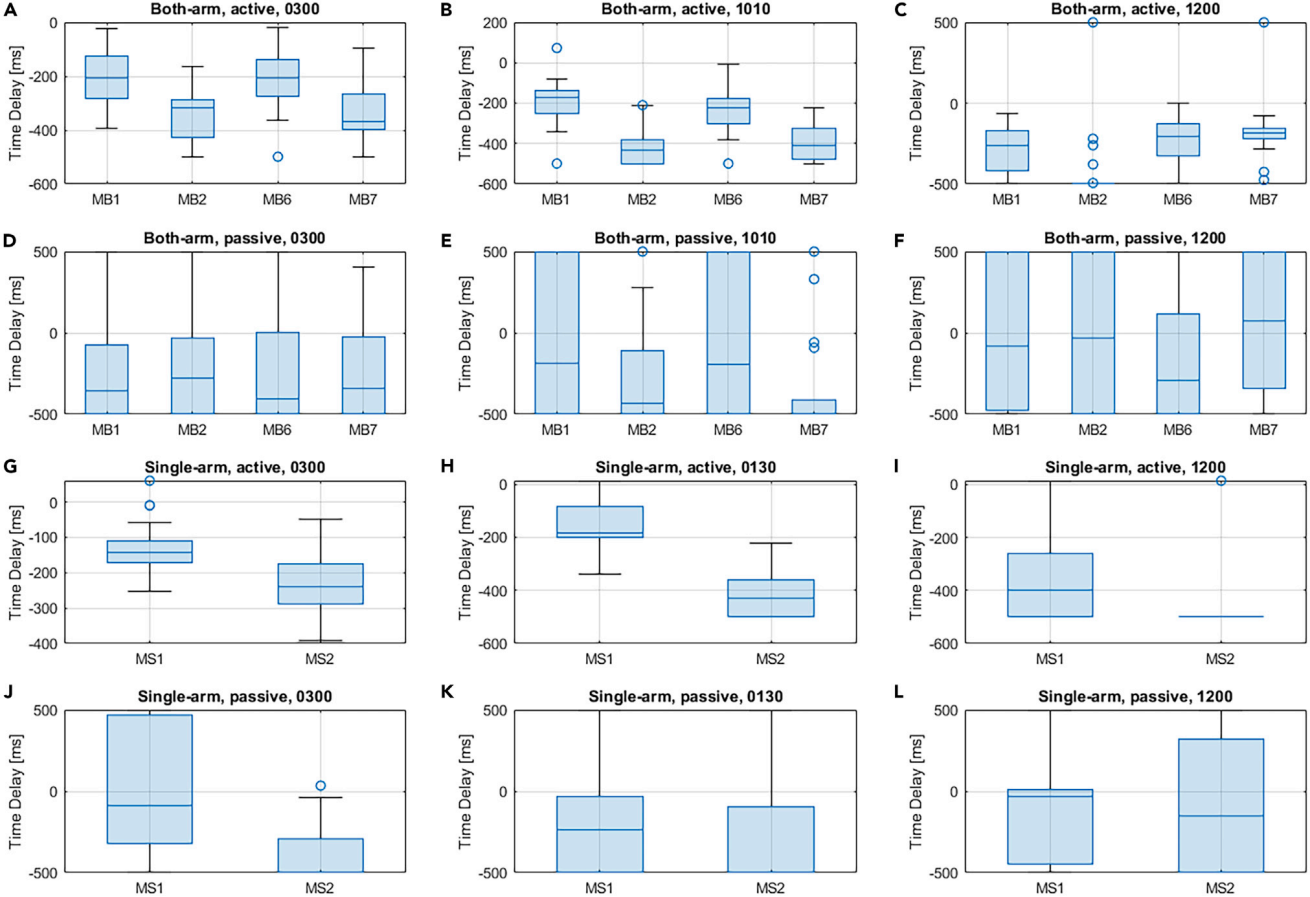
Statistics of the time delay between neuromuscular signals and the steering torque considering the four driving modes and the three hand positions. (A) The cross-correlation results of both-arm, active steering with hand position of 3 o'clock. (B) The results of both-arm, active steering with hand position of 10:10. (C) The results of both-arm, active steering with hand position of 12 o'clock. (D) The results of both-arm, passive steering with hand position of 3 o'clock. (E) The results of both-arm, passive steering with hand position of 10:10. (F) The results of both-arm, passive steering with hand position of 12 o'clock. (G) The cross-correlation results of single-arm, active steering with hand position of 3 o'clock. (H) The results of single-arm, active steering with hand position of 1:30. (I) The results of single-arm, active steering with hand position of 12 o'clock. (J) The results of single-arm, passive steering with hand position of 3 o'clock. (K) The results of single-arm, passive steering with hand position of 1:30. (L) The results of single-arm, passive steering with hand position of 12 o'clock. See also Tables S21–S24.

The statistical results of the time delay between the neuromuscular signals and the steering torque considering different steering tasks and hand positions are shown in Figure 5. Based on the results, for the both-arm active steering cases, it could be found that different hand positions could lead to different patterns of the time delay. For the both-arm active steering scenario, the muscle signals of the MB1, MB2, MB6, and MB7 of the both-arm arrangement showed significant lags compared with the steering torque with the three hand positions. Among the four muscles, the MB2 generated the largest time delays with the three hand positions, which were −335 ± 97 ms, −412 ± 95 ms, and −424 ± 221 ms. Considering the three hand positions for the both-arm active driving mode, the 10:10 o'clock hand position could lead to the largest mean time delay (−311 ± 105 ms) compared with the other two positions (−273 ± 102 ms for the 3 o'clock and −276 ± 170 ms for the 12 o'clock position). For the both-arm passive steering scenario, the four key muscles showed significant time delay to the steering torque when the hands were put in the 3 o'clock and 10:10 o'clock positions. For the 12 o'clock hand position, the muscle activity of MB1 showed a small relevant delay to the steering torque. In addition, both the MB2 and MB7 muscles showed positive delay (15 ± 457 ms and 62 ± 429 ms) to the steering torque. As illustrated in Figure 5, for the single-arm active steering, both the two key muscles, i.e., MS1 and MS2, showed a negative time delay to the torque signal. The 12 o'clock hand position resulted in the largest time delay among the three hand positions. An average value of the time delay of MS1 at −350 ± 158 ms among all the participants was observed, and an average value of 470 ± 121 ms was detected as the time delay of MS2 at that moment. For the single-arm passive steering scenario, MS1 showed a small negative time delay at −5 ± 386 ms with the hand position of 3 o'clock, whereas MS2 generated the largest time delay of −371 ± 177 ms.

### Smoothness Analysis of the Steering Activity

The steering smoothness, which was a key performance factor for driving, was analyzed using the steering torque signal. Two different approaches, namely, approximate entropy and the sliding standard deviation (SSD) test, were applied to evaluate the smoothness. Key results are reported as follows, and detailed statistics can be found in Tables S25–S32.

**Figure 6:**
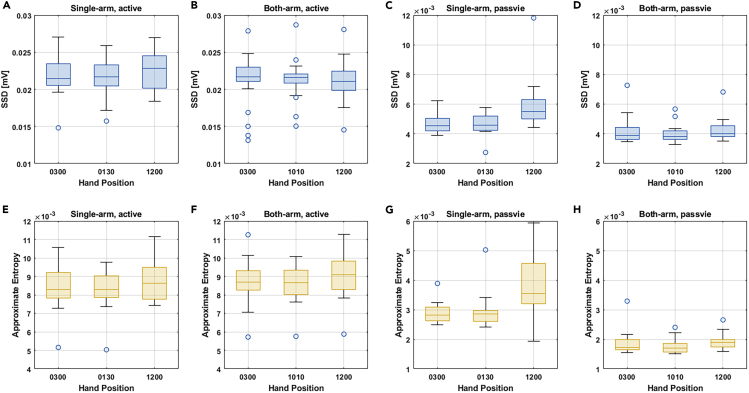
Statistical results for the steering smoothness evaluation with the steering torque signal Two different smoothness evaluation methods were applied: the sliding standard deviation (SSD) test and the approximate entropy test. The SSD tests for the different driving modes and hand positions are illustrated in the first row with red boxes. The approximate entropy test results are shown in the bottom row with blue boxes. (A) The results of single-arm, active steering under the SSD test. (B) The results of both-arm, active steering under the SSD test. (C) The results of single-arm, passive steering under the SSD test. (D) The results of both-arm, passive steering under the SSD test. (E) The results of single-arm, active steering under the approximate entropy test. (F) The results of both-arm, active steering under the approximate entropy test. (G) The results of single-arm, passive steering under the approximate entropy test. (H) The results of both-arm, passive steering under the approximate entropy test. See Tables S25–S32.

As shown in Figure 6, similar results were obtained with these two approaches. For the single-arm active steering, at the 1:30 o’clock position, the smoothest results were generated as 0.0084 ± 0.0014 by the approximate entropy method and 0.0219 ± 0.0027 by the SSD test approach. The worst smoothness performance was generated with the hand on the 12 o'clock position, and the results were 0.0090 ± 0.0016 when using the approximate entropy method and 0.0224 ± 0.0027 with the sliding SSD test. For the single-arm passive steering, the statistical results that were obtained based on the approximate entropy were similar to the regularity measurements at 3 o'clock and 1:30 o'clock positions. According to the sliding SSD test results, the 1:30 o'clock position led to a smoother control performance than the 3 o'clock position. In terms of the both-arm active steering, the two algorithms showed consistent results in that the performance at the 3 o'clock position was slightly smoother than that at the 10:10 o'clock position. The smoothness and regularity measurement using the approximate entropy method generated the results of 0.0087 ± 0.0012 for the 3 o'clock position and 0.0086 ± 0.0009 for the 1:30 o'clock position. Similarly, the SSD test-based approach generated the result of 0.0210 ± 0.0035 for the 3 o'clock position and 0.0213 ± 0.0026 for the 10:10 o'clock position. Then for both-arm passive steering, the hand position of 10:10 led to a slightly smoother performance than the 3 o'clock position. Last, it was interesting to see that for both single-arm and both-arm steering, the 12 o'clock position led to the worst performance in terms of the steering smoothness.

## Discussion

For the both-arm steering experiments, based on the correlation analysis, it could be found that in the active mode, the muscle correlation patterns at the 3 o'clock and 10:10 o'clock hand positions were similar. The activities of the pectoralis major of the clavicular portion and the deltoid anterior muscles, i.e., MB1, MB2, MB6, and MB7, showed strong correlations to the steering behavior in both the clockwise and counterclockwise directions. A steering direction-dependent result was observed in the passive steering experiments. Specifically, the left pectoralis major of the clavicular portion and the deltoid anterior muscles, i.e., MB6 and MB7, were strongly correlated with the active steering in the clockwise direction. The right pectoralis major and deltoid anterior muscles, i.e., MB1 and MB2, were strongly correlated with the active steering in the counterclockwise direction. The steering activities at the 12 o'clock position generated different patterns compared with the others. The right deltoid anterior muscle, i.e., MB2, did not show a strong correlation to the active steering that was shown in the 3 o'clock and 10:10 o'clock cases. Based on the both-arm experiment results, it could be found that the muscle correlations had consistent patterns for both the active and passive steering maneuvers. The limb muscles played different roles in the steering activities for different hands positions. The correlation analysis for hands on the 3 o'clock and 10:10 o'clock positions led to similar results, which indicated that the two distinct hand positions required a similar pattern of neuromuscular responses. However, the 12 o'clock positions showed a significantly different pattern to the other two postures. Similar results can be drawn from other parts of this study, which showed that the 12 o'clock position was quite different from the other two positions, whereas for the 3 o'clock and 10:10 o'clock positions, the neuromuscular dynamics of the upper limb muscles were similar due to the similar driving postures.

For the single-arm experiments, in the active steering mode, different correlation results were observed with the three different hand positions. Specifically, at the 3 o'clock position, the first three muscles showed significantly larger correlations than the others did. However, the correlation distributions became complex for the other two positions. For the 1:30 o'clock position, despite the first three muscles, the muscles MS5, MS9, and MS10 also showed strong correlations to the steering torque output. Last, for the tests with the hand position of 12 o'clock, only the MS1, MS4, and MS9 muscles showed strong correlations to the steering torque. Based on the aforementioned results mentioned, it could be concluded that single-arm active steering was very sensitive to the hand positions. Different hand positions during steering activities led to different patterns of muscle responses. The correlation distributions of the single-arm arrangement for passive steering were also direction dependent, which was similar to the patterns of the both-arm passive steering scenarios. The first three key muscles showed strong correlations to the steering in the counterclockwise direction, whereas the resultant correlations during steering in the clockwise direction with the hand positions of 3 o'clock and 1:30 o'clock were very small. For the 12 o'clock position, the top three key muscles, i.e., MS1, MS2, and MS4, also showed significantly high correlations to the steering in counterclockwise direction but low relations to the clockwise steering.

Based on the experiment results of the both-arm and single-arm steering, it could be found that the correlation patterns of the neuromuscular dynamics were consistent in different directions when performing active steering. However, the correlation patterns became direction sensitive when performing passive steering. Among all the measured muscles, according to the amplitude and contribution analysis, the pectoralis major of the clavicular portion and the deltoid anterior were the two most important muscles for steering maneuvers. According to the amplitude analysis, the patterns were different for different hand positions when the drivers performed active steering with the single-arm arrangement. Specifically, the hand position of 3 o'clock led to the largest values in terms of amplitude, whereas at the 12 o'clock position, the amplitudes reached their lowest values. Based on this observation, when performing single-arm active steering, the 12 o'clock position was a more energy-efficient position compared with the other position. Although the results of the passive steering for the 1:30 o'clock hand position showed an inverse trend, there was no significant difference among the three hand positions. For both-arm steering, the lowest average amplitude was found when both hands were in the 10:10 o'clock position. However, there was also no significant difference between these positions in both active and passive steering. In summary, when performing active steering, the most energy-efficient manner that led to the lowest average value of the amplitude was the type of single-arm steering with the 12 o'clock hand position. For active steering using both arms, the most energy-efficient hand position was the 10:10 o'clock position.

According to the time delay analysis, it was found that different muscles showed significantly different delays to the steering torque. In particular, during both-arm active steering, the right pectoralis major of the clavicular portion (MB1), the right deltoid anterior muscle (MB2), the left pectoralis major of the clavicular portion (MB6), and the left deltoid anterior muscle (MB7) showed consistent patterns of a significantly negative time delay to the steering torque. This indicated that the activations of these muscles caused the variation of the steering torque. For the both-arm passive steering experiments, the right deltoid anterior muscle (MB2) and the left deltoid anterior muscle (MB7) showed positive time delay to the steering torque at the 12 o'clock position. For the single-arm active steering, the right pectoralis major of the clavicular portion (MS1) and the deltoid anterior muscle (MS2) showed consistent patterns of a negative time delay to the steering torque at any hand position. Among all the testing cases, single-arm active steering with a hand position of 12 o'clock led to the largest time delay for the steering torque left after the neuromuscular signals.

In addition, based on the analysis of the smoothness of the driving performance, it was observed that when performing a single-arm active steering maneuver, for the 1:30 o'clock hand position, a smoother steering performance could be generated compared with the results at other two positions. When performing both-arm active steering, a smoother performance could be achieved at the 3 o'clock position. There was no significant difference found between the 3 o'clock and the 1:30 o'clock (in the single-arm case) positions or the 10:10 o'clock position (in the both-arm case) when performing passive steering. For either the both-arm or the single-arm driving modes, the 12 o'clock position always generated the worst control performance among the three positions in terms of smoothness. Although the single-arm active steering with the hand at the 12 o'clock position could generate the lowest average value of neuromuscular amplitudes, it also led to a larger variation and unstable steering performance compared with the other two postures. Finally, although the mean values of the smooth metrics for the both-arm steering were smaller than those for the single-arm steering, there was no significant difference between the two steering modes. Considering the aforementioned observations in the smoothness analysis, to ensure smooth, stable, and efficient driving, it was recommended to perform steering control using a single arm in the 1:30 o'clock position or using both hands in the 3 o'clock and 10:10 o'clock positions.

### Limitations of the Study

Two main limitations of this study are as follows. First, the driving environment and driving tasks in the study were simulated, which were not exactly the same as those in the real-world driving scenarios. The designed driving tasks for the participants may not fully reflect the naturalistic driving behaviors, especially under critical and emergency driving situations. Second, the participants involved in this study were all young male drivers, and their neuromuscular dynamics can be different from elderly and female drivers. Therefore, future works can be carried out from the following two aspects: more participants with diverse ages, gender, and driving habits can be recruited to do the test and real vehicle experiments with driver EMG signal measurement can be designed and conducted to collect real-world data for further analysis and validation.

### Resources Availability

#### Lead Contact

Further information and requests should be directed to and will be fulfilled by the Lead Contact: Chen Lv (lyuchen@ntu.edu.sg).

#### Materials Availability

This study did not generate new unique materials.

#### Data and Code Availability

The data and code that support all the findings of this study are available from the corresponding authors upon reasonable request.

## Methods

All methods can be found in the accompanying Transparent Methods supplemental file.

## Supplemental Information

Document S1. Transparent Methods, Figures S1 and S2, and Tables S1–S32

## References

[bib1] Abbink D.A., Mulder M., Van Paassen M.M. (2011). October. Measurements of muscle use during steering wheel manipulation. 2011 IEEE International Conference on Systems, Man, and Cybernetics.

[bib2] Abbink D.A., Mulder M., Van der Helm F.C., Mulder M., Boer E.R. (2011). Measuring neuromuscular control dynamics during car following with continuous haptic feedback. IEEE Trans. Syst. Man, Cybern. B (Cybern.).

[bib3] Bansal P., Kockelman K.M., Singh A. (2016). Assessing public opinions of and interest in new vehicle technologies: an Austin perspective. Transp. Res. Part C Emerg. Technol..

[bib4] Bi L., Wang M., Wang C., Liu Y. (2015). Development of a driver lateral control model by integrating neuromuscular dynamics into the queuing network-based driver model. IEEE Trans. Intell. Transp. Syst..

[bib5] Eriksson A., Stanton N.A. (2017). Takeover time in highly automated vehicles: noncritical transitions to and from manual control. Hum. Factors.

[bib6] Erlien S.M., Fujita S., Gerdes J.C. (2015). Shared steering control using safe envelopes for obstacle avoidance and vehicle stability. IEEE Trans. Intell. Transp. Syst..

[bib7] Fan X., Zhao C., Chen X., Jiang Y., Shen Y., Shen T. (2019). July. Review of the research on car seating comfort. International Conference on Applied Human Factors and Ergonomics.

[bib8] Fuchs S., Rass S., Lamprecht B., Kyamakya K. (2007). July. Context-awareness and collaborative driving for intelligent vehicles and smart roads. 1st International Workshop on ITS for an Ubiquitous ROADS.

[bib9] Hallé S., Chaib-draa B. (2005). A collaborative driving system based on multiagent modelling and simulations. Transportation Res. Part C: Emerging Tech..

[bib10] Hayama R., Liu Y., Ji X., Mizuno T., Kada T., Lou L. (2013). Preliminary research on muscle activity in driver’s steering maneuver for driver’s assistance system evaluation. Proceedings of the FISITA 2012 World Automotive Congress.

[bib11] Hoult W., Cole D.J. (2008). A neuromuscular model featuring co-activation for use in driver simulation. Vehicle Syst. Dyn..

[bib12] Kyriakidis M., Happee R., de Winter J.C. (2015). Public opinion on automated driving: results of an international questionnaire among 5000 respondents. Transp. Res. F traffic Psychol. Behav..

[bib13] Liu Y., Liu Q., Lv C., Zheng M., Ji X. (2017). A study on objective evaluation of vehicle steering comfort based on driver's electromyogram and movement trajectory. IEEE Trans. Hum. Mach. Syst..

[bib14] Lv C., Cao D., Zhao Y., Auger D.J., Sullman M., Wang H., Dutka L.M., Skrypchuk L., Mouzakitis A. (2017). Analysis of autopilot disengagements occurring during autonomous vehicle testing. IEEE/CAA J. Automatica Sinica.

[bib15] Lv C., Wang H., Cao D., Zhao Y., Auger D.J., Sullman M., Matthias R., Skrypchuk L., Mouzakitis A. (2018). Characterization of driver neuromuscular dynamics for human–automation collaboration design of automated vehicles. IEEE/ASME Trans. Mechatron..

[bib16] National Highway Traffic Safety Administration (2017).

[bib17] Nash C.J., Cole D.J. (2016). Development of a novel model of driver-vehicle steering control incorporating sensory dynamics. Dyn. Veh. Roads Tracks.

[bib18] Nguyen A.T., Sentouh C., Popieul J.C. (2017). Sensor reduction for driver-automation shared steering control via an adaptive authority allocation strategy. IEEE/ASME Trans. Mechatron..

[bib19] Nunes A., Reimer B., Coughlin J.F. (2018). People must retain control of autonomous vehicles. Nature.

[bib20] Pick A.J., Cole D.J. (2006). Neuromuscular dynamics in the driver–vehicle system. Vehicle Syst. Dyn..

[bib21] Pick A.J., Cole D.J. (2007). Driver steering and muscle activity during a lane-change manoeuvre. Vehicle Syst. Dyn..

[bib22] Pick A.J., Cole D.J. (2008). A mathematical model of driver steering control including neuromuscular dynamics. J. dyn. Syst. Meas. Control.

[bib23] Saleh L., Chevrel P., Claveau F., Lafay J.F., Mars F. (2013). Shared steering control between a driver and an automation: stability in the presence of driver behavior uncertainty. IEEE Trans. Intell. Transp. Syst..

[bib24] Smith P.H. (1936). Who's A good driver?. Sci. Am..

[bib25] Xing Y., Lv C., Wang H., Cao D., Velenis E. (2020). An ensemble deep learning approach for driver lane change intention inference. Transp Res. Part C Emerg. Technol..

