# Supplementary material for: Pattern Recognition and Characterization of Upper Limb Neuromuscular Dynamics during Driver-Vehicle Interactions
Source: iScience. 2020 Sep 7;23(9):101541. doi: 10.1016/j.isci.2020.101541 (PMC7509210; doi:10.1016/j.isci.2020.101541)
Supplement: Document S1. Transparent Methods, Figures S1 and S2, and Tables S1–S32 [file mmc1.pdf]

iScience, Volume *23*

Supplemental Information

Pattern Recognition and Characterization of Upper Limb Neuromuscular Dynamics during Driver-Vehicle Interactions

Yang Xing, Chen Lv, Yifan Zhao, Yahui Liu, Dongpu Cao, and Sadahiro Kawahara

Related to Figure 1. Supplemental Figures

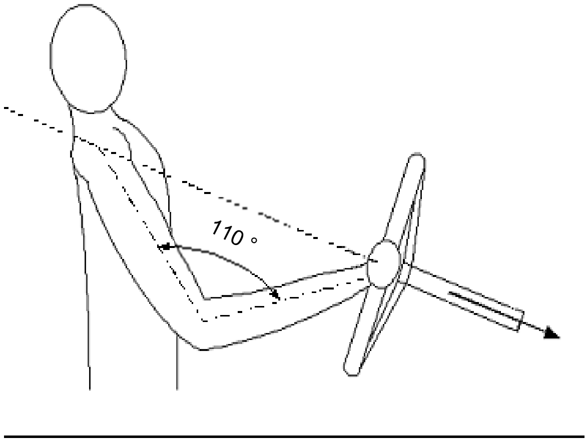

Figure S1. The requirement of the basic posture of a test subject during experiments. Related to Figure 1. The participants are required to seat in the simulator and steer the wheel as usual. At beginning of test, the test participants must hold the steering wheel at 3 o' clock position with the right hand. The test participants' arm is slightly bent at the elbow where the forearm and upper arm form an angle about 100-110deg. And the steering wheel is raked so that a projected line along the steering axis is parallel to a line through the shoulder and wrist joint. During passive test or fixed steering wheel test, participants posture should be kept constant as the initial posture. During active steering test, participants should steer mostly in the tangential direction to the wheel. The participants must be familiar with the operation before measurement.

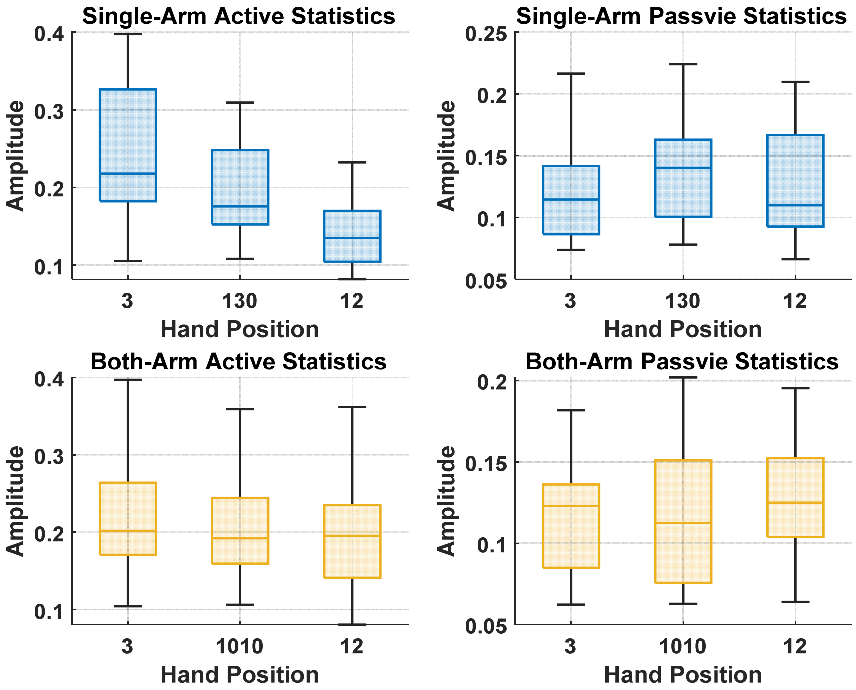

Figure S2. Overall amplitude statistics of the participants regarding the four driving modes and three kinds of hand positions. Related to Figure 3. The summations of ten EMG signals are first calculated for each participant. Then, the statistics of the overall amplitude for each hand position of the driving mode are determined accordingly. The upper two boxplots are statistic results for the single-arm active and passive mode, while the bottom two boxplots are statistic results for the both-arm active and passive mode, respectively. Based on the single-arm experiment, it can be found that the single-arm active steering with the 3-clock position generates the largest overall amplitude of the ten muscles (0.242±0.092 mv) while the single-arm active steering with hand on the 12-clock position shows the lowest overall amplitude (0.142±0.044 mv). Although the single-arm active steering with the 3-clock position shows the largest muscle amplitude, the corresponding passive steering mode generates the lowest overall amplitude among the three modes (0.121±0.038 mv). At this moment, the single passive steering with hand on 130-clock position leads to the highest amplitude (0.136±0.043). Regarding both-arm active steering maneuver, although the test with hands on the 3-clock position generates the largest overall amplitude (0.217±0.076), there is no significant difference exists between the three positions. A similar trend can be found in the both-arm passive steering mode, where the mean amplitude of all the participants is 0.117±0.034 with hands on the 3-clock position, 0.117±0.045 with hands on the 10-10-clock position, and 0.126±0.037 with hands on the 12-clock position.

Supplemental Tables

Signals measurement and sensors in the experiments

Table S1. EMG signals measurement and sensors requirements in the tests. Related to Figure 1. Signals measurement includes EMG signals, steering angle, steering torque. Sensors include: electromyograph, steering torque and angle sensor.

Detected muscles	Sensitivity/Max/Sample frequency/ Unit
Pectoralis Major of Clavicular portion	0.5mV/5mV/1000Hz/mV
Anterior Deltoid	0.5mV/5mV/1000Hz/mV
Middle Deltoid	0.5mV/5mV/1000Hz/mV
Posterior Deltoid	0.5mV/5mV/1000Hz/mV
Triceps Long head	0.5mV/5mV/1000Hz/mV
Triceps Lateral head Exterior	0.5mV/5mV/1000Hz/mV
Infraspinatus	0.5mV/5mV/1000Hz/mV
Biceps	0.5mV/5mV/1000Hz/mV
Pectoralis Major of Sternal portion	0.5mV/5mV/1000Hz/mV
Teres Major	0.5mV/5mV/1000Hz/mV

Table S2. Torque and angle measurement and sensors requirements. Related to Figure 1.

Detected signals	Sensitivity/Max/Sample frequency/ Unit
Steering torque	4/20/1000Hz/N·m
Steering angle	36/180/1000Hz/deg

Cross-correlation statistics

Table S3. Cross-correlation statistics for the both-arm clockwise active steering. Related to Figure 2. Related to Figure 2.

Positive	Metric	PM R	DA R	DP R	TLH R	TM R	PM L	DA L	DP L	TLH L	TM L
0300	Mean	0.7610	0.8574	0.6204	0.6124	0.7076	0.8195	0.8764	0.6448	0.6144	0.6869
	SD	0.0874	0.0642	0.0535	0.0774	0.0905	0.0682	0.0950	0.0958	0.0542	0.0947
1010	Mean	0.8027	0.8377	0.6216	0.6452	0.7583	0.8273	0.8459	0.6450	0.6428	0.7301
	SD	0.0678	0.0604	0.0442	0.0583	0.1121	0.0675	0.0755	0.0931	0.0572	0.0857
1200	Mean	0.8335	0.6813	0.6709	0.6658	0.7541	0.8158	0.8024	0.6810	0.6569	0.6866
	SD	0.0749	0.1426	0.0792	0.0728	0.0867	0.0715	0.0847	0.0597	0.0917	0.0709

Table S4. Cross-correlation statistics for the both-arm counterclockwise active steering. Related to Figure 2.

Negative	Metric	PM R	DA R	DP R	TLH R	TM R	PM L	DA L	DP L	TLH L	TM L
0300	Mean	0.7660	0.8733	0.5996	0.5890	0.6838	0.7775	0.8382	0.6053	0.5847	0.6581
	SD	0.1036	0.0523	0.0621	0.0796	0.0897	0.0655	0.0874	0.1047	0.0549	0.1006
1010	Mean	0.7971	0.8265	0.5844	0.6063	0.7301	0.7900	0.8036	0.6028	0.6044	0.7100
	SD	0.0804	0.0647	0.0526	0.0718	0.1136	0.0617	0.0740	0.0975	0.0597	0.1020
1200	Mean	0.8348	0.6537	0.6463	0.6377	0.7387	0.8226	0.8147	0.6558	0.6365	0.6624
	SD	0.0804	0.1395	0.0765	0.0843	0.0878	0.0866	0.0888	0.0787	0.0989	0.0681

Table S5. Cross-correlation statistics for the both-arm clockwise passive steering. Related to Figure 2.

Positive	Metric	PM R	DA R	DP R	TLH R	TM R	PM L	DA L	DP L	TLH L	TM L
0300	Mean	0.3732	0.2636	0.5660	0.6173	0.5863	0.8098	0.8523	0.6680	0.5946	0.6480
	SD	0.1134	0.0994	0.0270	0.0423	0.0640	0.0767	0.0818	0.0718	0.0566	0.0930
1010	Mean	0.3537	0.2600	0.6053	0.6322	0.6285	0.7953	0.8431	0.5986	0.5751	0.5940
	SD	0.1235	0.1035	0.0900	0.1069	0.1317	0.1225	0.0832	0.1183	0.0625	0.1409
1200	Mean	0.2362	0.3157	0.7429	0.6639	0.6344	0.3754	0.4842	0.6665	0.5952	0.6338
	SD	0.0886	0.1289	0.1129	0.0883	0.0486	0.1095	0.1412	0.0663	0.0095	0.0773

Table S6. Cross-correlation statistics for the both-arm counterclockwise passive steering. Related to Figure 2.

Negative	Metric	PM R	DA R	DP R	TLH R	TM R	PM L	DA L	DP L	TLH L	TM L
0300	Mean	0.7791	0.8648	0.6373	0.5814	0.6412	0.3503	0.2797	0.5245	0.5943	0.5678
	SD	0.0962	0.0706	0.0403	0.0289	0.0583	0.1074	0.1140	0.0986	0.0623	0.0920
1010	Mean	0.7660	0.8399	0.5778	0.5491	0.5860	0.3494	0.2579	0.5747	0.6250	0.6085
	SD	0.1261	0.0819	0.0854	0.0797	0.1255	0.1274	0.1080	0.1534	0.0652	0.1287
1200	Mean	0.8654	0.7786	0.4775	0.5472	0.5655	0.7814	0.7065	0.5339	0.5957	0.5601
	SD	0.0514	0.0901	0.1178	0.0791	0.0476	0.0739	0.1275	0.0682	0.0132	0.0580

Table S7. Cross-correlation statistics for the single-arm clockwise active steering. Related to Figure 2.

Positive	Metric	PM R	DA R	DM R	DP R	TLH R	TLH R	BS R	INFS R	PM R	TM R
0300	Mean	0.8146	0.8560	0.8292	0.6710	0.7707	0.6898	0.7727	0.7216	0.7444	0.7569
	SD	0.0539	0.0294	0.0622	0.0698	0.1056	0.0606	0.0768	0.0514	0.0941	0.1130
0130	Mean	0.8665	0.7715	0.6985	0.6461	0.7756	0.6563	0.6111	0.7254	0.8214	0.7992
	SD	0.0548	0.0653	0.0751	0.0737	0.1135	0.0866	0.0721	0.0984	0.0706	0.1154
1200	Mean	0.7597	0.5699	0.5473	0.7726	0.6971	0.6063	0.5769	0.6251	0.7941	0.6989
	SD	0.2023	0.1735	0.1842	0.2163	0.1966	0.1648	0.1669	0.1845	0.2079	0.1861

Table S8. Cross-correlation statistics for the single-arm counterclockwise active steering. Related to Figure 2.

Negative	Metric	PM R	DA R	DM R	DP R	TLH R	TLH R	BS R	INFS R	PM R	TM R
0300	Mean	0.8776	0.9325	0.8954	0.7215	0.7674	0.7418	0.8407	0.7625	0.7467	0.7616
	SD	0.0501	0.0241	0.0541	0.0800	0.0846	0.0553	0.0591	0.0643	0.0703	0.0774
0130	Mean	0.9227	0.8204	0.7443	0.6666	0.7743	0.6946	0.6236	0.7545	0.8654	0.7857
	SD	0.0428	0.0850	0.0799	0.0881	0.0822	0.0829	0.0986	0.1181	0.0701	0.0926
1200	Mean	0.8660	0.6437	0.6365	0.8152	0.7499	0.6804	0.6419	0.7155	0.8980	0.7563
	SD	0.0583	0.1147	0.1165	0.0873	0.0698	0.0831	0.1240	0.1124	0.0579	0.0828

Table S9. Cross-correlation statistics for the single-arm clockwise passive steering. Related to Figure 2.

Positive	Metric	PM R	DA R	DM R	DP R	TLH R	TLH R	BS R	INFS R	PM R	TM R
0300	Mean	0.2477	0.1646	0.3416	0.5770	0.6751	0.5483	0.2907	0.4197	0.5825	0.5584
	SD	0.0920	0.0516	0.1141	0.1078	0.1213	0.0608	0.1084	0.1171	0.0781	0.1412
0130	Mean	0.2115	0.2140	0.3663	0.6915	0.6992	0.5004	0.3780	0.5091	0.5189	0.5848
	SD	0.1489	0.1601	0.1395	0.1511	0.1281	0.1073	0.1578	0.0855	0.0860	0.1468
1200	Mean	0.2132	0.2414	0.5555	0.8295	0.7137	0.6102	0.6576	0.4821	0.4032	0.6766
	SD	0.2016	0.1845	0.1966	0.2005	0.1754	0.1585	0.1976	0.1558	0.1751	0.1690

Table S10. Cross-correlation statistics for the single-arm counterclockwise passive steering. Related to Figure 2.

Negative	Metric	PM R	DA R	DM R	DP R	TLH R	TLH R	BS R	INFS R	PM R	TM R
0300	Mean	0.8338	0.8854	0.8239	0.6459	0.5318	0.6536	0.8588	0.7283	0.6026	0.6882
	SD	0.0551	0.0423	0.0779	0.0898	0.1108	0.0602	0.0654	0.1154	0.0706	0.1304
0130	Mean	0.8545	0.8318	0.7861	0.5181	0.5303	0.6965	0.7769	0.6609	0.6689	0.6462
	SD	0.1077	0.1055	0.1015	0.1571	0.1222	0.1001	0.1226	0.0707	0.0937	0.1427
1200	Mean	0.8770	0.8105	0.6220	0.3456	0.5057	0.5967	0.5293	0.7068	0.7839	0.5407
	SD	0.1440	0.1464	0.1729	0.2172	0.1599	0.1456	0.2235	0.1370	0.1578	0.1627

Amplitude analysis of neuromuscular signals

Table S11. Statistics of the amplitudes of the EMG signals for the both-arm active steering. Related to Figure 3.

Active	Metric	PM R	DA R	DP R	TLH R	TM R	PM L	DA L	DP L	TLH L	TM L	Sum
0300	Mean	0.0508	0.0373	0.0059	0.0052	0.0075	0.0484	0.0387	0.0103	0.0053	0.0075	0.2171
	SD	0.0588	0.0501	0.0047	0.0044	0.0082	0.0571	0.0511	0.0116	0.0042	0.0115	0.2618
1010	Mean	0.0457	0.0326	0.0056	0.0053	0.0085	0.0440	0.0370	0.0105	0.0054	0.0082	0.2027
	SD	0.0559	0.0431	0.0044	0.0046	0.0118	0.0532	0.0465	0.0123	0.0050	0.0114	0.2480
1200	Mean	0.0478	0.0236	0.0067	0.0061	0.0110	0.0473	0.0345	0.0104	0.0057	0.0078	0.2008
	SD	0.0730	0.0320	0.0065	0.0055	0.0165	0.0639	0.0469	0.0123	0.0050	0.0094	0.2709

Table S12. Statistics of the amplitudes of the EMG signals for the both-arm passive steering. Related to Figure 3.

Passive	Metric	PM R	DA R	DP R	TLH R	TM R	PM L	DA L	DP L	TLH L	TM L	Sum
0300	Mean	0.0197	0.0209	0.0054	0.0053	0.0056	0.0201	0.0219	0.0069	0.0054	0.0057	0.1170
	SD	0.0240	0.0273	0.0041	0.0043	0.0046	0.0240	0.0279	0.0066	0.0067	0.0060	0.1354
1010	Mean	0.0182	0.0204	0.0053	0.0057	0.0060	0.0170	0.0216	0.0077	0.0057	0.0063	0.1140
	SD	0.0238	0.0266	0.0045	0.0051	0.0057	0.0209	0.0281	0.0079	0.0059	0.0072	0.1356
1200	Mean	0.0311	0.0202	0.0064	0.0063	0.0057	0.0287	0.0125	0.0055	0.0050	0.0052	0.1266
	SD	0.0424	0.0244	0.0062	0.0056	0.0048	0.0320	0.0130	0.0047	0.0044	0.0055	0.1432

Table S13. Statistics of the amplitudes of the EMG signals for the single-arm active steering. Related to Figure 3.

Active	Metric	PM R	DA R	DM R	DP R	TLH R	TLH R	BS R	INFS R	PM R	TM R	Sum
0300	Mean	0.0725	0.0517	0.0146	0.0068	0.0092	0.0095	0.0175	0.0215	0.0173	0.0159	0.2365
	SD	0.0893	0.0760	0.0196	0.0062	0.0118	0.0093	0.0211	0.0249	0.0236	0.0234	0.3052
0130	Mean	0.0658	0.0414	0.0112	0.0066	0.0087	0.0084	0.0093	0.0141	0.0174	0.0135	0.1965
	SD	0.1000	0.0586	0.0136	0.0063	0.0104	0.0087	0.0098	0.0168	0.0247	0.0208	0.2697
1200	Mean	0.0418	0.0191	0.0086	0.0109	0.0081	0.0069	0.0085	0.0079	0.0141	0.0160	0.1418
	SD	0.0640	0.0281	0.0103	0.0151	0.0087	0.0058	0.0082	0.0085	0.0221	0.0227	0.1933

Table S14. Statistics of the amplitudes of the EMG signals for the single-arm passive steering. Related to Figure 3.

Passive	Metric	PM R	DA R	DM R	DP R	TLH R	TLH R	BS R	INFS R	PM R	TM R	Sum
0300	Mean	0.0260	0.0335	0.0105	0.0062	0.0060	0.0073	0.0135	0.0091	0.0066	0.0086	0.1273
	SD	0.0340	0.0469	0.0124	0.0053	0.0059	0.0064	0.0163	0.0101	0.0062	0.0090	0.1525
0130	Mean	0.0340	0.0335	0.0129	0.0073	0.0069	0.0088	0.0116	0.0067	0.0070	0.0085	0.1373
	SD	0.0476	0.0473	0.0157	0.0082	0.0069	0.0087	0.0137	0.0062	0.0069	0.0090	0.1702
1200	Mean	0.0315	0.0227	0.0110	0.0110	0.0074	0.0088	0.0101	0.0063	0.0088	0.0082	0.1258
	SD	0.0491	0.0327	0.0114	0.0152	0.0076	0.0081	0.0105	0.0065	0.0105	0.0077	0.1591

Table S15. Statistics of the average amplitudes for the both-arm steering maneuver. Related to Figure 3.

Both-Arm	Mean	SD
Active 3	0.2171	0.0757
Passive 3	0.1170	0.0341
Active 10-10	0.2027	0.0695
Passive 10-10	0.1140	0.0439
Active 12	0.2008	0.0709
Passive 12	0.1266	0.0359

Table S16. Statistics of the average amplitudes for the single-arm steering maneuver. Related to Figure 3.

Single-Arm	Mean	SD
Active 3	0.2365	0.0869
Passive 3	0.1274	0.0403
Active 130	0.1965	0.0638

Passive 130	0.1373	0.0645
Active 12	0.1418	0.0441
Passive 12	0.1258	0.0459

Overall contribution analysis of neuromuscular signals

Table S17. Overall muscle contribution statistics for both-arm active steering. Related to Figure 4.

Active	Metric	PM R	DA R	DP R	TLH R	TM R	PM L	DA L	DP L	TLH L	TM L
0300	Mean	0.2230	0.2019	0.0233	0.0204	0.0327	0.2144	0.1929	0.0395	0.0206	0.0313
	SD	0.0707	0.0532	0.0083	0.0079	0.0111	0.0670	0.0593	0.0689	0.0068	0.0103
1010	Mean	0.2261	0.1827	0.0229	0.0230	0.0408	0.2108	0.1897	0.0406	0.0233	0.0401
	SD	0.0791	0.0523	0.0073	0.0082	0.0166	0.0652	0.0578	0.0710	0.0078	0.0150
1200	Mean	0.2551	0.1100	0.0304	0.0278	0.0561	0.2383	0.1748	0.0487	0.0253	0.0344
	SD	0.1015	0.0763	0.0156	0.0128	0.0377	0.0887	0.0831	0.0588	0.0104	0.0102

Table S18. Overall muscle contribution statistics for both-arm passive steering. Related to Figure 4.

Passive	Metric	PM R	DA R	DP R	TLH R	TM R	PM L	DA L	DP L	TLH L	TM L
0300	Mean	0.1608	0.1892	0.0453	0.0454	0.0480	0.1688	0.1925	0.0568	0.0446	0.0486
	SD	0.0800	0.0639	0.0156	0.0225	0.0167	0.0608	0.0689	0.0536	0.0160	0.0169
1010	Mean	0.1465	0.1798	0.0489	0.0523	0.0584	0.1391	0.1963	0.0679	0.0521	0.0589
	SD	0.0735	0.0553	0.0228	0.0253	0.0330	0.0607	0.0652	0.0626	0.0260	0.0266
1200	Mean	0.2580	0.1610	0.0534	0.0495	0.0437	0.2174	0.0940	0.0437	0.0389	0.0404
	SD	0.1083	0.0688	0.0228	0.0174	0.0141	0.0850	0.0699	0.0156	0.0141	0.0124

Table S19. Overall muscle contribution statistics for single-arm active steering. Related to Figure 4.

Active	Metric	PM R	DA R	DM R	DP R	TLH R	TLH R	BS R	INFS R	PM R	TM R
0300	Mean	0.3005	0.2346	0.0630	0.0272	0.0412	0.0375	0.0738	0.0844	0.0705	0.0674
	SD	0.0834	0.0461	0.0244	0.0096	0.0217	0.0122	0.0341	0.0300	0.0260	0.0348
0130	Mean	0.3479	0.2092	0.0520	0.0297	0.0472	0.0375	0.0379	0.0743	0.0919	0.0724
	SD	0.0889	0.0490	0.0267	0.0094	0.0200	0.0110	0.0110	0.0470	0.0293	0.0271
1200	Mean	0.3052	0.1119	0.0503	0.0865	0.0605	0.0460	0.0498	0.0565	0.1149	0.1184
	SD	0.0899	0.0402	0.0288	0.0326	0.0241	0.0136	0.0169	0.0280	0.0342	0.0406

Table S20. Overall muscle contribution statistics for single-arm passive steering. Related to Figure 4.

Passive	Metric	PM R	DA R	DM R	DP R	TLH R	TLH R	BS R	INFS R	PM R	TM R
0300	Mean	0.2009	0.2886	0.0805	0.0451	0.0446	0.0506	0.1148	0.0654	0.0455	0.0640
	SD	0.0815	0.0580	0.0297	0.0136	0.0185	0.0214	0.0555	0.0241	0.0130	0.0147
0130	Mean	0.2602	0.2548	0.0869	0.0496	0.0507	0.0636	0.0834	0.0442	0.0479	0.0586
	SD	0.0780	0.0490	0.0297	0.0152	0.0224	0.0374	0.0361	0.0182	0.0150	0.0160
1200	Mean	0.2942	0.1910	0.0711	0.0892	0.0543	0.0599	0.0659	0.0479	0.0695	0.0569
	SD	0.0792	0.0499	0.0207	0.0379	0.0211	0.0242	0.0125	0.0195	0.0236	0.0113

Time delays analysis

Table S21. Time delays statistics for the both-arm active steering [ms]. Related to Figure 5.

Active	Metric	PM R	DA R	DP R	TLH R	TM R	PM L	DA L	DP L	TLH L	TM L
0300	Mean	-209	-335	-196	-131	40	-213	-334	-205	-120	-164
	SD	104	97	252	330	366	111	96	191	151	184
1010	Mean	-202	-412	217	2	-168	-234	-395	-208	-145	-129
	SD	128	95	432	294	81	108	87	207	230	232
1200	Mean	-284	-424	-412	-31	-147	-224	-174	-364	-119	-69
	SD	146	221	171	281	171	137	176	131	106	183

Table S22. Time delays statistics for the both-arm passive steering [ms]. Related to Figure 5.

Passive	Metric	PM R	DA R	DP R	TLH R	TM R	PM L	DA L	DP L	TLH L	TM L
0300	Mean	-232	-207	2	-43	1	-227	-281	-110	-45	7
	SD	354	319	414	413	391	347	262	378	420	320
1010	Mean	-101	-257	-28	26	-164	-65	-327	-73	191	-66
	SD	419	331	450	432	345	423	338	353	403	407
1200	Mean	-12	15	-88	49	-71	-156	62	-76	-108	54
	SD	410	457	370	435	404	380	429	372	432	381

Table S23. Time delays statistics for the single-arm active steering [ms]. Related to Figure 5.

Active	Metric	PM R	DA R	DM R	DP R	TLH R	TLH R	BS R	INFS R	PM R	TM R
0300	Mean	-129	-225	-159	-199	-71	-54	-267	-230	43	-38
	SD	77	91	104	126	72	185	126	205	76	145
0130	Mean	-156	-419	-396	-46	-38	-168	-397	-210	-57	-55
	SD	92	85	129	386	154	228	230	252	80	146
1200	Mean	-350	-470	203	473	144	162	403	-306	-189	48
	SD	158	121	406	118	351	373	190	185	87	246

Table S24. Time delays statistics for the single-arm passive steering [ms]. Related to Figure 5.

Passive	Metric	PM R	DA R	DM R	DP R	TLH R	TLH R	BS R	INFS R	PM R	TM R
0300	Mean	-5	-371	185	167	77	185	49	342	246	180
	SD	386	177	316	324	295	411	312	300	309	283
0130	Mean	-182	-252	-6	-73	14	-113	35	66	162	88
	SD	345	344	343	235	358	354	350	302	298	343
1200	Mean	-115	-86	-240	-93	88	-5	-63	-39	-94	-95
	SD	344	400	348	285	286	300	325	381	339	282

Smoothness analysis of the steering performance

Table S25. Statistics of the regularity with SSD for the single-arm active steering. Related to Figure 6.

Active	Mean	STD
0300	0.0217	0.0027
0130	0.0215	0.0027
1200	0.0224	0.0027

Table S26. Statistics of the regularity with SSD for the both-arm active steering. Related to Figure 6.

Active	Mean	STD
0300	0.0210	0.0035
1010	0.0213	0.0026
1200	0.0211	0.0027

Table S27. Statistics of the regularity with SSD for the single-arm passive steering. Related to Figure 6.

Passive	Mean	STD
0300	0.0047	0.0007
0130	0.0046	0.0007
1200	0.0065	0.0028

Table S28. Statistics of the regularity with SSD for the both-arm passive steering. Related to Figure 6.

Passive	Mean	STD
0300	0.0042	0.0009
1010	0.0040	0.0005
1200	0.0043	0.0007

Table S29. Statistics of the regularity with Approximate Entropy for single-arm active steering. Related to Figure 6.

Active	Mean	STD
0300	0.0086	0.0017
0130	0.0084	0.0015
1200	0.0090	0.0016

Table S30. Statistics of the regularity with Approximate Entropy for both-arm active steering. Related to Figure 6.

Active	Mean	STD
0300	0.0087	0.0012
1010	0.0086	0.0009
1200	0.0090	0.0011

Table S31. Statistics of the regularity with Approximate Entropy for single-arm passive steering Related to Figure 6.

Passive	Mean	STD
0300	0.0029	0.0004
0130	0.0030	0.0006
1200	0.0053	0.0046

Table S32. Statistics of the regularity with Approximate Entropy for both-arm passive steering. Related to Figure 6.

Passive	Mean	STD
0300	0.0018	0.0004
1010	0.0018	0.0003
1200	0.0019	0.0002

Supplemental Information

Transparent Methods

Experiment Design

A human-in-the-loop driving simulator, as shown in Figure 1, was used as a test rig for a range of steering experiments. The test subjects were required to sit on the driver's seat of the driving simulator, which included a driver's cab for a passenger vehicle with a steering system. The reaction torque actuator that was equipped in the simulator could generate steering torque based on the calculation results of the vehicle model in its controller. The basic posture of a test subject is shown in Figure S1. The seat and the steering wheel were adjusted to ensure that the upper limb was slightly bent at the elbow (approximately 110° between the forearm and upper arm). The line along the steering axis was approximately parallel to the line through the shoulder and wrist joints when the right arm was held at the 3 o'clock position and when the left arm was held at the 9 o'clock position. The basic posture of the test subject was approximately the same in the driving environment, and the basic posture was easy to maneuver in the experiment. Two steering tasks were designed, as shown in the subplots (g) and (f) in Figure 1, including Task A, a steering test with a single arm, i.e., the right arm, and Task B, a steering test with the both-arm arrangement. The detailed experiment description and the setup are introduced below. The neuromuscular dynamics were measured by EMG signals in mV, and the steering activities were measured by driver's steering torque in N·m and steering angle in degree, respectively. Detailed specifications of the measured signals can be found in Table S1 and Table S2.

1) Task A: Steering test with a single-arm arrangement

The purpose of Task A was to study the relationship between the EMG signals of the single arm (i.e., the right arm) and the steering torque applied to the hand wheel during naturalistic driving. Two testing scenarios, namely the passive steering and active steering, were designed. The active steering is the major and most common driver-vehicle-interaction manner for conventional and low-level automated driving vehicles. The neuromuscular dynamics of the driving during active steering reflect how drivers use their upper limb muscles to control the steering wheel and steadily control the vehicle. While, the passive steering reflects how driver response to the external disturbance torque, which can be generated by the road situation, vehicle vibration or the driver assistance system. It can also reflect how driver's intent differs from the automation's decision in automated vehicles. Therefore, it is also needed to analyze the driver passive steering maneuver to better understand the driver-automation interactions.

Passive steering with a single arm: The passive steering task was set to mimic the steering maneuver, which restrained the steering angle movement against the external disturbance torque for lane keeping or vehicle stabilization in naturalistic driving. In the experiment, the subject was asked to keep the steering wheel at the neutral position using a single arm, i.e., the right arm, when the external disturbance torque was given by the driving simulator. The external disturbance torque was given by a triangle wave with a constant frequency of 0.025 Hz and an amplitude of 5 Nm, as shown in Figure 1g. The amplitude value was selected with reference to the steering torque value in the normal driving of a vehicle equipped with a power steering system. The frequency value was chosen in a semi-static level in order to research the preliminary activities of a muscle.

Active steering with a single arm: The active steering task was set to mimic the steering maneuver for entering a corner and returning from a corner to straight line driving in naturalistic driving. In this experiment, the subject was asked to hold the steering wheel fixed with the required hand posture in the beginning, and then start to steer the hand wheel with the required angular position using their right arm. The required angular position was a sinusoidal angle input with a constant frequency of 0.25 Hz and an approximate amplitude of 60°, as shown in Figure 1f.

2) Task B: Steering test with both-arm arrangement

The purpose of Task B was to study the relation between the EMG signals of both arms and the steering torque applied to the hand wheel during naturalistic driving. The passive steering and the active steering that were used in the single-arm driving test were also adopted.

Passive steering with both-arm: In this experiment, the subject was asked to keep the steering wheel at the neutral position using two arms when the external disturbance torque was given by the driving simulator. The external disturbance torque was given by a triangle wave with a constant frequency of 0.025 Hz and an amplitude of 5 Nm, as shown in Figure 1g. The amplitude value was selected with reference to the steering torque value in the normal driving of a vehicle equipped with a power steering system. The frequency value was chosen at a semi-static level in order to research the preliminary activities of the muscles.

Active steering with both-arm: In this experiment, the subject was asked to hold the steering wheel fixed with the requiring hand posture in the beginning, and then start to steer the hand wheel with the required angular position using both arms. The required angular position was a sinusoidal angle input with a constant frequency of 0.025 Hz and an amplitude of 60°, as shown in Figure 1f.

3) Hand postures

Both-arm with three hand positions: In this scenario, the driver used the steering wheel with both arms. There were three gripping positions on the steering wheel, which were the 3 o'clock, 10:10 and 12 o'clock positions. The 3 o'clock position as well as the 12 o'clock position indicated that the test subjects were required to grasp the steering wheel at the locations of the above times using two hands. For the 10:10 position, the left and right hands of the subject were placed at the locations of the hour and ten past the hour on a clock, respectively. A schematic diagram of the above hand positions is illustrated in Figure 1c (1)-(3). Each test subject completed both the active and passive steering tasks for all six hand positions.

Single-arm only with three hand positions: In this scenario, the driver operated the steering wheel using the right arm only, and the left hand is held away from the steering wheel. The three gripping positions were the 3 o'clock position, 12 o'clock position, and 1:30 o'clock position, which correspond to Figure 1c (4), (5), and (6), respectively. The test subjects were required to grasp the steering wheel at the locations of the hour hand, which pointed to the above-mentioned times, in order to complete the driving tasks.

4) Muscle selection and EMG signal measurement

In order to measure a muscle electromyography (EMG) signal (Lv *et al.*, 2018) that could reflect the muscle behavior during a steering maneuver, it was necessary to determine the key muscles that were involved in the steering maneuver to generate steering torque. The study in (Liu *et al.*, 2017) has indicated some key muscles for this, including the anterior deltoid, pectoralis clavicular, pectoralis sternal, posterior deltoid, middle deltoid, and triceps long head. It has also been indicated that the muscles of the shoulder are likely to be important for generating steering torque. These results were used here. Furthermore, not only the extension and flexion of the upper arm that were around the shoulder joint but also abduction, adduction, supination, and pronation were included based on kinesiology. Thus, addition to the muscles described above, the biceps, teres major, and infraspinatus were measured in this experiment. In the single-arm steering scenarios, ten different neuromuscular signals, denoted as MS1-MS10, were measured from the right upper limb. These ten signals were the pectoralis major of the clavicular portion, the deltoid anterior, the deltoid middle (lateral), the deltoid posterior, the triceps long head, the triceps lateral head exterior, the biceps, the infraspinatus, the pectoralis major, and the teres major. For the steering experiments with the both-arm arrangement, ten EMG signals, denoted as MB1-MB10, were detected from both the right and left upper limbs. These signals were the pectoralis major of clavicular portion, the deltoid anterior, the deltoid posterior, the triceps long head, and the teres major of the right arm and the left arm. The electrode placements for the EMG measurement are shown in Figure 1d.

Participants

In total, 42 subjects in the age range of 22-50 (31±7) years were recruited for the experiments. Each subject had a valid driving license. All of the subjects had no previous knowledge of the research topic. Among these participants, 20 were randomly assigned to conduct the single-arm experiments, and the rest of the 22 participants were engaged in the both-arm steering experiments. The study protocol and consent form were approved by the JTEKT Corporation, Japan, and consent was obtained from all subjects.

Correlation Analysis

The cross-correlation analysis was used to measure the correlation between the ten measured neuromuscular signals and the applied steering torque during normal driving. The best match between the two signals could also be determined using cross-correlation. In this study, three levels of the correlation strength, namely, the strong correlation, moderate correlation, and weak correlation, were defined. Additionally, all the correlation statistics were bounded within the range of 0-1. The value of the correlation between 0.75 and 1 indicated a strong correlation. The value of the correlation between 0.3 and 0.75 indicated a moderate correlation, and the correlation value that was positioned between 0.3 and 0 indicated a weak correlation. The cross-correlations for some muscles were direction-dependent, which meant that the correlations between some neuromuscular signals and the steering torque were sensitive to the steering directions (the clockwise and counterclockwise directions). Therefore, the cross-correlations were calculated separately. A moving average filter with a span of five data points was applied in order to smooth the EMG signals before calculating the cross-correlation. The cross-correlation was calculated with the MATLAB function in the statistical analysis toolbox. The correlation and the time delays of the two time-series signals could then be determined accordingly. To initiate the cross-correlation function, the maximum lag was selected as 3000, and the cross-correlation sequence was normalized.

Amplitude Analysis

This section describes the analysis of the amplitudes of the EMG signals for the ten different muscles. The amplitudes of the EMG signals reflected the signal strengths and to what extent the specific muscles influenced and determined the steering maneuvers. Due to the co-contraction mechanism, it was essential to analyze the muscle activation patterns under different driving scenarios with distinguished hand postures according to the amplitude of the EMG signals. Hence, the amplitudes of the EMG signals were analyzed based on two aspects, which were directional-oriented and non-directional oriented. Lastly, an integrated weighted muscle contribution analysis was proposed based on the joint consideration of the correlation and directional-oriented amplitudes.

1) Directional-Oriented and Non-Directional-Oriented Amplitude Analysis

The aim of the directional-oriented amplitude analysis was to provide a clear visualization of the different patterns of the EMG signals in different steering directions (i.e., the clockwise and counterclockwise directions). Three different analysis approaches were adopted using muscle strength features. First, the muscle strengths were statistically analyzed with respect to different hand postures. The strength of each EMG signal was statistically analyzed with a boxplot and bar chart. The muscle strengths for different participants were identified separately based on the steering direction. Then the statistical visualization, including the median and SD of the directional-oriented muscle amplitude, could be described with the boxplots. Second, the total muscle strength for the single-arm and both-arm steering scenarios was compared. For each participant, the total muscle strength was the summation of the amplitudes of all ten measured muscles with consideration of the directions. Then the mean value of the total muscle strengths for each driving mode for all participants was compared with respect to different hand positions. Third, the mean value of the muscle strength for each muscle was also compared with respect to different steering modes in order to understand the different patterns of the muscle amplitudes for different driving scenarios.

A similar analysis process was proposed for the non-directional oriented analysis. For the no-direction-oriented cases, the absolute value of the steering torque without consideration of the steering direction was used. The non-direction-oriented amplitude analysis was the summation of the amplitudes of the measured muscles without consideration of the directions. The analysis generated a direct visualization of the different distributions of the muscle strengths with respect to the different driving modes and muscles.

2) Weighted Muscle Contribution Analysis

A weighted muscle contribution analysis was proposed based on the integration of the correlation and the amplitude of each EMG signal, for which the correlation was used as the weight of the amplitude. The purpose of setting this weighted muscle contribution index was to identify the most relevant muscles while eliminating the less important muscles used in the steering tasks. For example, although the teres major muscle showed a strong correlation based on the cross-correlation analysis, the absolute amplitude of its signal was very weak. This meant that the teres major muscle was not a determinative muscle. The weighted muscle contribution was calculated based on the direction-dependent correlation and amplitude

of each EMG signal. Specifically, the correlation and amplitude of each EMG signal with respect to the counterclockwise and clockwise directions were multiplied separately at first, and then the contributed muscle importance was the summation of the generated products of both directions. The contributed energy E_i of each muscle could first be given with Eq. (1):

$$E_i = Corr\_pos_i \times Amp\_pos_i + Corr\_neg_i \times Amp\_neg_i, \quad (1)$$

where E_i is the contribution index of the ith muscle, and $Corr\_pos_i$ and $Corr\_neg_i$ are the correlation values of the ith muscle with respect to the positive steering torque (i.e. in clockwise direction) and the negative steering torque (i.e. in counterclockwise direction), respectively. Amp_pos_i and Amp_neg_i are their corresponding muscle amplitudes. The overall contribution of the certain muscle $Cont_i$ among all the measured muscles could then be represented as:

$$Cont_i = \frac{E_i}{\sum_{i=1}^{10} E_i} \quad (2)$$

Based on the proposed contribution assessment method, the most determinative muscles could be better identified and visualized as the importance, which represented a joint consideration of both the correlations and the absolute strengths of the EMG signals.

Steering Smoothness Analysis

It was important to maintain smooth steering operation in our naturalistic driving in order to ensure the ride comfort and vehicle safety. In this study, the smoothness of each participant's steering action was evaluated based on two different methods, namely, the approximate entropy method and the sliding window method.

1) Approximate Entropy Method

Because the participants were required to steer the hand wheel and to generate a steering torque according to the experimental requirements, the expected steering torque could be seen as a determinative and predictable sequence. Therefore, the regularity and the variation of the output torque could be used to evaluate the smoothness and the stability of the steering performance. The approximate entropy (ApproxEnt), which was an efficient solution for the regularity measurement of the non-linear time-series signals, was used (Pincus, 1991). A delayed reconstruction $Y_{1:L}$ with the same length (N points) of the original steering torque sequence X with a lag of τ was generated at first. Then the numbers of the points within the range at each sampling step N_i could be determined as (Pincus, 1991)

$$N_i = \sum_{i=1.i\neq k}^{L} \mathbf{1}(\|Y_i - Y_k\|_\infty < R), \quad (3)$$

where $\mathbf{1}(\cdot)$ is the indicator function, and R is the radius of the similarity.

Then the approximate entropy of the sequence could be calculated as

$$ApproxEnt = \Phi_m - \Phi_{m+1}, \quad (4)$$

where

$$\Phi_m = (N - m + 1)^{-1} \sum_{l=1}^{N-m+1} \log(N_i). \quad (5)$$

The smaller the value of the approximate entropy of a certain sequence, the more regular and stable the system is. The approximate entropy of each sequence was calculated with the MATLAB built-in function. The embedding dimension of the function was two, the reconstruction lag was one, and the radius of similarity was selected as $0.2 \times variance(X)$, where X is the steering torque sequence.

2) Sliding Window Method

Another method that was adopted in order to measure the signal's irregularity and smoothness was the sliding window approach. In this method, a sliding window was used to calculate the standard deviation of the sliding slice. Then the average STD of the signal over the entire time horizon was statistically analyzed based on the SD of these segments. To increase the computational efficiency, a non-overlapped sliding window method was used. The sliding window size in this study was selected as 100 data points, which

was a 100 ms segment. The STD index (STDI) of the smoothness of the entire steering period could be represented by the mean STD of the whole sliding slices of each sequence:

$$STDI = \frac{1}{N}\sum_N \sqrt{\frac{\sum_{m=1}^{M}(x_m - \mu_M)^2}{M}} \tag{6}$$

where N is the total number of the sliding slices of the entire sequence, M is the selected window size for each slice, x_m is the mth point within the segment, and μ_m is the mean value of the segment.

The size of the sliding window was 100 points, which was small enough to smoothly evaluate the stability of each slice. It should be noted that a sliding window of a very large size could not efficiently measure the local smoothness of the steering torque, which could lead to a coarse statistical result. However, the size of the sliding window could not be too small because a single window could only provide a short period variation, and a fixed single window would be unable to measure the dynamic trends and significant variations among the signals.

Statistical Methods

The statistical analysis was performed in Matlab (R2019b, MathWorks) and Microsoft Excel. The statistical significance was determined using paired t tests at the α = 0.01 threshold level throughout the research. The central tendency was estimated using the mean.

SUPPLEMENTAL REFERENCES

[In addition to references already cited in the main paper]

Pincus, S.M., 1991. Approximate entropy as a measure of system complexity. *Proceedings of the National Academy of Sciences*, 88(6), pp.2297-2301.
